# The Care of Rheumatic Children

**Published:** 1903-11-07

**Authors:** 


### Hospital Clinics and Medical Progress.

### THE CARE OF RHEUMATIC CHILDREN.

It is within the experience of every practitioner that many children who come under observation with undeniable evidences of organic heart disease present a personal record entirely free from recognised rheumatic incidents. Careful questioning may in some instances elicit a history of vague pains in the joints or of one or more attacks of tonsillitis, but even when these are admitted they are evidently regarded as events of no moment, and if the question of rheumatism or rheumatic fever is raised in set terms it is met with an unqualified negative. To the physician familiar with the manifestations of rheumatism in early life all this presents no difficulty. It is due, as he knows, to the fact that the popular conception of rheumatism is based upon the symptoms of the disease as these are displayed in the adult. In the view of the public, rheumatism is an affection of the joints, which, in its more acute forms, is apt to be attended by some form of heart disease. But to the physician this is a very limited and inadequate presentation of the disease. Whilst fairly accurate so far as rheumatism in the adult is concerned, it is seriously defective when the disease as it occurs in childhood is taken into consideration. Here an acute polyarthritis is only an occasional occurrence, and the clinical picture, though less definite, comprises a much larger and more varied series of events. Inflammatory conditions of the joints occupy an entirely subordinate position, and such manifestations as tonsillitis, purpura, erythema, chorea, subcutaneous nodules and pleurisy come into prominence. Further, it is certain that with any one of these, pericarditis or endocarditis may be, and indeed frequently is, associated. From these facts it is but a short step to the proposition that disease of the heart may be the sole clinical consequence of an attack of rheumatism. Whether this proposition is or is not accepted it is beyond dispute that such disease is often the most definite, as it is the most serious and persistent result of rheumatism in childhood. The other incidents are often so slight that even if they attract attention at the time the memory of them soon fades. It is in the order of facts thus briefly presented that an explanation of the existence of cardiac valvular disease in individuals who deny all history of rheumatism is to be found. The denial is inaccurate because the definition is defective.

The contrast between the phenomena of rheumatism in the adult and the disease as seen in children may be reasonably held to account for the greater frequency of cardiac complications at the earlier age. An acute polyarthritis necessarily drives the sufferer, whether he will or no, to practise a policy of complete rest. But the child who is the victim of rheumatism in its more subtle forms largely escapes this compulsion. For the adult, therefore, an acute or sub-acute attack of rheumatism means inevitably the adoption of measures which lessen the strain on the cardio-vascular mechanism, whilst many of the phases of rheumatism in childhood are consistent with continued bodily activity, and thus the patient unconsciously maintains the very conditions which favour permanent damage to the cardiac valves.

If this contention is well founded there emerges from it a manifest indication of the direction from which an improved state of affairs may be anticipated. Let it once be appreciated that children with a rheumatic inheritance are liable to suffer from slight and inconspicuous evidences of the disease; that with each of these there is a definite risk of cardiac complications; that the most certain method of avoiding such complications is absolute rest in bed until all disturbances have subsided—and there is a fair prospect of obtaining for these children the protective influence under which the adult shelters himself as a matter of necessity. At present the slight rheumatic manifestations which are so frequent in children are largely disregarded because their serious significance and the dangers they entail are not appreciated. The parents dismiss them as trifling disorders which require little or no attention. Experience, moreover, often appears to justify this attitude. The child with slight pains in his joints or a sore throat seems to be well in a day or two, and hence similar attacks in the same or in other members of the family are met in an equally light-hearted fashion. It is only when some chance circumstance leads to a medical examination, perhaps years after the mischief has been done, that the discovery is made of the existence of organic disease of the cardiac valves.

It is manifestly impossible to prevent these unfortunate developments unless the parents of rheumatic children are made alive to the dangerous risks which are involved in even the slight illnesses of the younger members of the family. The parental view of rheumatism, in short, must be brought up to the level of current professional opinion. Such a process of education can be conducted only by the individual practitioner. It rests with him, as opportunity offers, to instruct the parents and in this way to protect the children. The opportunity presents itself whenever events show the practitioner that he is accepting professional responsibility for the welfare of a family whose disease proclivities are of a rheumatic order. Then is his chance, or rather then is his duty, to speak in no uncertain tones regarding the dangers which attend the slighter illnesses and complaints of the younger members of the household. His responsibility is not restricted to the cure of the individual patient. It has a wider and possibly even a more beneficent range. With him it rests by the utterance of a timely warning to secure in advance the prompt adoption of the early and complete rest which of all known measures is the one best calculated to protect young children against the dangers and penalties of organic cardiac disease. Everyone allows that in the presence of infectious disease the protection of others is a part of the practitioner's obvious duty. The suggestion now advanced is that an equal claim arises from a diagnosis of rheumatism—that the recognition of a rheumatic strain in any family is a circumstance which demands such instruction of the heads of the household as will stimulate them to take the steps necessary to protect their children from the most serious possibility of the tendency these inherit. Were this rule universally practised it is not unreasonable to anticipate that the occurrence of endocarditis might be considerably diminished.

Good advice, it is true, is not always valued or adopted. None the less is it the duty of those possessed of knowledge to warn others of the approach of danger. The prevention of disease is one of the most attractive functions of the profession. Here is an opportunity which may be realised on a large scale and which calls for no more elaborate mechanism than insight, good judgment, and tact.

